# Proximate and biochemical characterization of burrito (*Bachydeuterus auritus*) and flying gurnard (*Dactylopterus volitans*)

**DOI:** 10.1002/fsn3.401

**Published:** 2016-06-21

**Authors:** Lawrence D. Abbey, Mary Glover‐Amengor, Margaret Ottah Atikpo, Nazlin K. Howell

**Affiliations:** ^1^ Food Research InstituteP. O. Box M 20AccraGhana; ^2^ School of Biological and Molecular SciencesUniversity of SurreyGuildfordSurreyGU2 5XHUnited Kingdom

**Keywords:** Amino acid, polyunsaturated fatty acids, underutilized fish

## Abstract

With limited protein resources and depleting commercial fish species there is the need to improve utilization of some of the lesser known species which are underutilized, for example, big eye grunt (burrito), *Bachydeuterus auritus,* and the flying gurnard (*Dactylopterus volitans*), (other names Cephalocanthus volitans (local) *Pampansre*). This study was to characterize some of the proximate and biochemical properties of burrito and the flying gurnard so as to evaluate their potential for use in human nutrition and other value‐added products. Proximate and chemical analysis were determined by the methods of AOAC. Fatty acid profiles were determined following the method of Saaed and Howell (1999). Amino acid profiles for the species were determined according to Bidlingmeyer et al. (1987). The protein content of both the water soluble and salt soluble protein extracts of the fish species were determined by the Bradford Protein Assay method (Bradford 1976). Rancidity of the fish species was assessed by thiobarbituric acid reactive substances (TBARS) and Peroxide value (PV) as described by Saeed and Howell (1999). Burrito contained 18% protein, whereas the flying gurnard contained 22.3%. Calcium content was 296 mg/100 g for burrito and 185 mg/100 g for flying gurnard, whereas iron content was 4.1 mg/100 g and 1.0 mg/100 g for burrito and the flying gurnard, respectively. Palmitic acid (C16) was 27% and 14.3% for the flying gurnard and burrito, respectively. C17: 1ω8 was 3% in the flying gurnard and 0.2% in burrito. Oleic (C18:1ω9) was 17% in the flying gurnard and 6% in burrito. C20:4ω6 was 1.6% in the flying gurnard and 3% in burrito. Docosahexaenoic acid (C22:6ω3) was 4.9% in the flying gurnard and 4.0% in burrito. Both burrito and the flying gurnard are of high nutritional quality as they had a high protein content, good general amino acid profile and abundance of polyunsaturated fatty acids.

## Introduction

The promotion and utilization of underutilized or low‐priced fish species such as small pelagics and other by‐catch for human consumption have intensified rapidly globally to meet the ever‐increasing demand for protein especially in a developing country like Ghana. Fish and fishery products account for the major animal protein intake in the diet of the majority of Ghanaians, as these are the cheapest animal protein available. Fish provides about 60% of the country's protein requirement (Mensah 1991). With limited resources, depleting commercial species due to several factors, there is the need to improve utilization of some of the lesser known species (Whittle and Wood 1992). Although underutilized pelagic species have no particularly desirable characteristics yet, the nutritional value of these fish is considered to be as high as that of any of the more desirable species in terms of protein quality and other nutrients like fats, notably the omega‐3 polyunsaturated fatty acids, minerals and vitamins (Nettleton 1985; Pigott and Tucker 1990).

Two of the lesser known and hence underutilized species are as follows: big eye grunt, *Bachydeuterus auritus* (Valenciennes, 1831), and the flying gurnard (*Dactylopterus volitans*) *(Linnaeus*, 1758) (other names Cephalocanthus volitans (local) *Pampansre*). This may be due to, among other factors, their dark flesh, bony nature and consumer prejudices against these species.

The big eye grunt which is popularly called burrito with local vernacular names including “*Moi*,” “*Boeboe*,” and “*Ebie*” is one of the most important by‐catch fish species in Ghanaian coastal waters. The flying gurnard is quite abundant in Ghanaian waters but due to its very low market value it is regarded as of no statistical importance (Anon 2001).

This study was to characterize some of the proximate and biochemical properties of burrito and the flying gurnard so as to evaluate their potential for use in human nutrition and other value‐added products.

## Materials and Methods

### Materials

Freshly harvested burrito (*Bachydeuterus auritus*) and the flying gurnard (*Dactylopterus volitans*) were obtained from the fishing harbor (August, 2012) in Tema, Ghana. They were immediately held in ice at 0°C and thereafter frozen at −30°C until analyzed. The fatty acid profile and protein profiling by the SDS–polyacrylamide gel electrophoresis were performed on myofibril proteins extracted from the muscles of frozen and thawed fish samples at the University of Surrey, in Guildford, United Kingdom. Determination of the proximate and chemical characterization was carried out at the Food Research Institute, Ghana.

### Methods

Protein, moisture, fat, ash, phosphorus, calcium, and iron were determined by the methods of AOAC (2016). The myofibril proteins were extracted from the fish muscles. Two grams of the fish muscle was homogenized with 25 mL of solution A (50 nmol/L phosphate buffer (pH 7.5) made up 1.25 g of sodium dihydrogen orthophosphate and 5.96 g of disodium hydrogen orthophosphate in a liter of water). The mixture was centrifuged at 5000*g* for 10 min. The supernatant was kept as the water soluble proteins. The pellet was resuspended in 30 mL of solution B (50 nmol/L phosphate buffer +0.8mol/L NaCl (pH 7.5) made up of 1.25 g of sodium dihydrogen orthophosphate, 5.96 g of disodium hydrogen orthophosphate and 46.75 g NaCl in a liter of water). The mixture was homogenized at 4,180 g, for 3 min. This was washed out with 20 mL of the same solution and kept in a cold room for 2 h after which it was centrifuged at 5000*g* for 20 min. The supernatant was kept as the salt soluble proteins and together with the water soluble proteins, were used for the analyses of protein content, amino acid, and the electrophoretic profile of proteins from the fish species. Amino acid profiles for the species were determined according to Bidlingmeyer et al. (1987). The elution profile of the derivatized amino acid components at 254 nm were carried out with a PICO TAG Amino Acid Analysis System (Waters, Millipore Co. Milford, MA) in a Nova‐pack C18 column (Waters). The protein content of both the water soluble and salt soluble protein extracts of the fish species were determined by the Bradford Protein Assay method (Bradford 1976). Standards were prepared from protein BSA. A 0.1 mL sample of unknown protein solution was added to 3 mL of Bradford reagent and vortexed immediately. The absorbance of the sample was read between 5–60 min at 595 nm in an Uvikon double‐beam spectrophotometer. The concentration was read from the standard curve. SDS–Polyacrylamide Gel electrophoresis of the myosin extracts of the species was by the method of Hames and Rickwood (1990) with a Phast Gel electrophoresis. Fat was obtained by cold extraction with chloroform and methanol from minced fish (Bligh and Dyer 1959) and was esterified as described by the method of Saeed and Howell (Saeed and Howell 1999) to determine their fatty acid profiles. Rancidity of the fish species was assessed by thiobarbituric acid reactive substances (TBARS) and Peroxide value (PV) as described by Saeed and Howell (1999).

### Statistical analysis

Excel spreadsheet was used to calculate means and standard deviations.

## Results

The protein contents of burrito and flying gurnard are shown in Table 1. Burrito contained 18% protein, whereas the flying gurnard contained 22.3%. The ash contents were 2% for burrito and 3.3% for the flying gurnard. Calcium content was 296 mg/100 g for burrito and 185 mg/100 g for flying gurnard, whereas iron content was 4.1 mg/100 g and 1.0 mg/100 g for burrito and the flying gurnard, respectively.

**Table 1:** Proximate and chemical compositions of Fresh burrito (*Bachydeuterus auritus*) and the flying gurnard (*Dactylopterus volitans)*

Parameter evaluated	Species	
	Burrito	The flying gurnard
Moisture% (wet weight basis)	80.8 ± 3.5	74.0 ± 2.5
Protein% (*N* × 6.25)	18 ± 4.3	22.3 ± 3.4
Fat %	0.6 ± 2.6	0.7 ± 1.8
Ash %	2 ± 0.8	3.3 ± 0.7
Calcium (mg/100 g)	296.0 ± 8	185.0 ± 11
Iron (mg/100)	4.1 ± 0.4	1.0 ± 0.5
Phosphorus(mg/100)	254.0 ± 17	215.6 ± 12

Values are means of three determinations ± standard deviation.

Protein concentration in the extracts of burrito and the flying gurnard are shown in Table 2. Burrito contained 4.6 mg/mL water soluble proteins and 6.3 mg/mL salt soluble proteins. The flying gurnard also contained 3.7 mg/mL water soluble proteins and 8.9 mg/mL salt soluble proteins.

**Table 2:** Protein concentration of protein extracts of burrito and the flying gurnard (mg/mL)

Fish Species	Water soluble proteins	Salt soluble proteins
Burrito	4.6 ± 1.2	6.3 ± 1.3
The flying gurnard	3.7 ± 1.4	8.9 ± 1.5

Values are means of three determinations ± standard deviation

**Figure 1:**
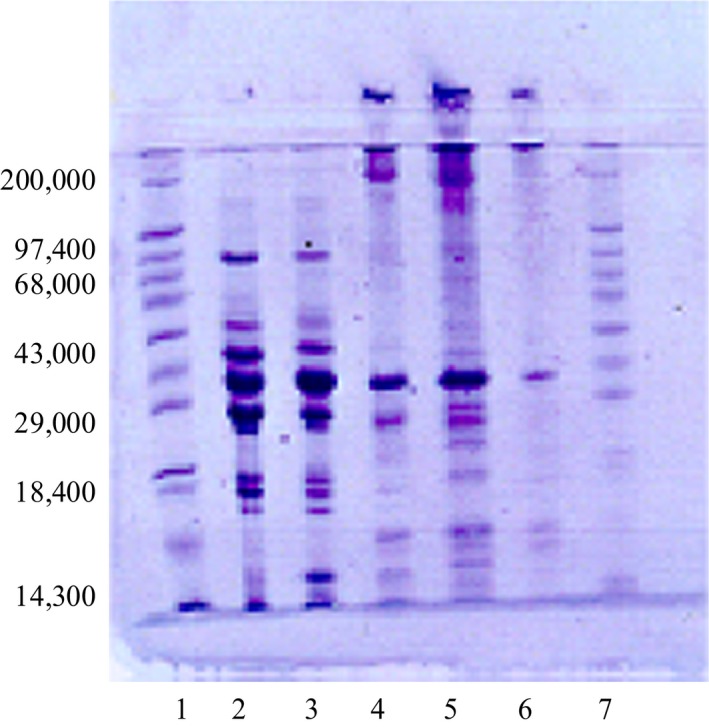
SDS–Polyacrylamide gel electrophoresis (SDS) patterns for The flying gurnard and Burrito*. (1 and 7) Standard, (2) Flying gurnard – WS (3) Burrito – WS (4) Burrito – SS, (5) Flying gurnard – SS, (6) Horse mackerel – SS. WS – Water soluble protein. SS – Salt soluble protein.

The electrophoretogram for burrito and the flying gurnard is shown in Figure 1.

The amino acid composition of burrito and the flying gurnard are shown in Table 3.

**Table 3:** Amino acid compositions of flying gurnard and burrito

Amino acids	BurritoSalt soluble proteins %	The flying gurnardSalt soluble proteins%
asp	6.6 ± 0.21	6.05 ± 0.45
glu	10.39 ± 0.22	9.9 ± 0.22
h. pro	1.7 ± 0.05	1.39 ± 0.11
ser	4.63 ± 0.09	4.76 ± 0.13
gly	7.11 ± 0.05	6.98 ± 0.22
his	0.78 ± 0.05	0.71 ± 0.07
arg	6.24 ± 0.1	5.59 ± 0.04
thr	4.6 ± 0.007	4.88 ± 0.08
ala	11.8 ± 0.26	12.07 ± 0.17
pro	2.99 ± 0.26	3.46 ± 0.39
tyr	4.56 ± 0.34	4.48 ± 0.05
val	5.77 ± 0.1	5.9 ± 0.03
met	2.97 ± 0.1	3.21 ± 0.34
Cys	1.14 ± 0.09	1.0 ± 0.06
I leu	3.96 ± 0.05	4.31 ± 0.02
leu	8.04 ± 0.1	8.27 ± 0.1
phe	3.37 ± 0.06	3.34 ± 0.007
trp	6.0 ± 0.33	5.34 ± 0.09
lys	7.32 ± 0.14	8.31 ± 0.64

Aspartate content for burrito was 6.6% while that of the flying gurnard was 6.05%. Glutamine contents were 10.9% for burrito and 9.9% for the flying gurnard. Arginine was 6.24% for burrito and 5.59% for the flying gurnard.

The fatty acid profile of the flying gurnard and burrito are shown in Table 4. Palmitic acid (C16) was 27% and 14.3% for the flying gurnard and burrito, respectively. C17: 1ω8 was 3% in the flying gurnard and 0.2% in burrito. Oleic (C 18:1ω9) was 17% in the flying gurnards and 6% in burrito. C 20:4ω6 was 1.6% in the flying gurnard and 3% in burrito. Docosahexaenoic acid (C 22:6ω3) was 4.9% in the flying gurnard and 4.0% in burrito. The P/S ratio of the polyunsaturated and saturated fatty acids were 1.9 for flying gurnard and 2.5 for the burrito.

**Table 4:** Fatty acid profile of the flying gurnard and the burrito

Fatty acid	The flying gurnard (%)	Burrito (%)
C14	0.01 ± 0	0.9 ± 0.2
C14.1	0.1 ± 0.03	0.01 ± .01
C15	1.1 ± 0.1	0.2 ± 0.1
C16	27 ± 3.6	14.3 ± 2.4
C 16:1ω7	7 ± 1.6	1.8 ± 0.4
C17	2.4 ± 0.8	1 ± 0.2
C17: 1ω8	0.3 ± 0.1	0.2 ± 0.1
C 18:0	0.1 ± 0.05	7 ± 1.3
C 18:1ω9	15 ± 2.7	6 ± 1.5
C 18:2ω6	1.6 ± 0.3	1.1 ± 0.4
C 18:3ω3	0.5 ± 0.1	0.3 ± 0.1
C20	2 ± 0.9	0.4 ± 0.1
C20: 1ω9	4 ± 1.2	3.3 ± 1.1
C 20:2ω6	1.6 ± 0.6	3 ± 1.3
C 20:3ω6	0.6 ± 0.3	1 ± 0.6
C 20:4ω6	8.7 ± 1.4	4.0 ± 1.8
C20: 4ω3	1 ± 0.3	2 ± 1.1
C20: 5ω3	7.3 ± 1.6	5 ± 1.6
C21: 5ω3	4.3 ± 1.2	3 ± 1.9
C22:0	1.3 ± 0.7	2 ± 1.3
C22: 1ω11 + ω13	0.6 ± 0.2	5 ± 1.9
C 22:4ω6	2.7 ± 0.7	27 ± 5.9
C 22:5ω6	0.4 ± 0.1	1.1 ± 0.5
C22: 5ω3	2.6 ± 0.6	2 ± 0.9
C22: 6ω3	4.9 ± 1.1	4.0 ± 1.7
Total saturated	33.9 ± 4.7	25.8 ± 3.8
Total mono unsaturated	0.6 ± 0.02	5 ± 1.9
Total polyunsaturated	63.8 ± 6.3	64.5 ± 5.9
Total ω3 content	20.6 ± 3.7	16.3 ± 2.7
P/S ratio	1.9	2.5

Values are means of three determinations ± standard deviation.

The thiobarbituric acid reactive substances (TBARS) and Peroxide value (PV) of the flying gurnard and burrito are shown in Table 5. TBARS (mgMDA/kg) value for flying gurnard was 1.1, whereas that for burrito was 1.2. PV (mEq/kg fish) for the flying gurnard was 2.5 and that for burrito was 2.7.

**Table 5:** The thiobarbituric acid reactive substances (TBARS) and Peroxide value (PV) of the Flying gurnard and the burrito

Indicator	The flying gurnard	Burrito
TBARS (mgMDA/kg)	1.1 ± 0.5	1.2 ± 0.3
PV (mEq/kg fish)	2.5 ± 0.8	2.7 ± 0.9

Values are means of three determinations ± standard deviation.

## Discussion

Table 1 shows that the protein contents of burrito (*Bachydeuterus auritus*) (18%) and the flying gurnard (*Dactylopterus volitans*) (22.3%) are above the levels of 16 ± 3% reported for pelagic fish by (Windsor and Barlow (1981). This suggests that the two species could be good sources of protein and may be used in fish protein concentrate production or in food supplements (Windsor and Barlow 1981). The ash content for the species (2% for burrito and 3.3% for the flying gurnard) were within the range of 0.5–1.8% of wet weight for most other fish species (Sidwell 1981). In comparison to other species the fat contents of burrito (*Bachydeuterus auritus*) (0.6%) and the flying gurnard (*Dactylopterus volitans* (0.7%) were below the range 1–8% reported for other pelagic species (King and Poulter 1985) and may not be suitable for fish oil production (Urdahl 1992). The calcium contents of the two species (296 mg/100 g for burrito and 185 mg/100 g for the flying gurnard) though quite high were also below the values of 580 mg/100 g of calcium reported for other fish species (Sidwell 1981). The phosphorus contents of the species (296 mg/100 g and 215.6 mg/100 g for burrito and the flying gurnard, respectively) were within the average values of fillets, which ranged from 113‐to 350‐mg/100 g (Sidwell 1981; Teeny et al. 1984). Although the iron content of the species analyzed were significantly different (*P* > 0.05) from each other with burrito (*Bachydeuterus auritus*) having a value of 4.1 mg/100 g and the flying gurnard (*Dactylopterus volitans)* 1.0 mg/100 g, they are within a wider range 0.8 mg/100 g–373 mg/100 g reported for many other pelagics (Sidwell 1981; Teeny et al. 1984).

The major amino acids (Table 3) present in both species are glutamine (9–10%) and alanine (11–12%). The fish species are also good sources of lysine (7–8%). Their percentage sulfur amino acid, methionine (2–3%), compares favorably to other species (Garrow and James 1993). The overall profiles of the essential amino acids of the two species appear to suggest that the species have a high class protein comparable to that of the mammalian meat which contains high levels of lysine and histidine (FAO, 1962; Garrow and James 1993; Friedman 1996). The fish species may be good sources of protein supplement in infants' diets.

From the fatty acids profiles of the fish species (Table 4), differences were observed in their qualitative and quantitative compositions though both showed predominance of palmitic acid (C16:0), oleic (C 18:1ω9), C 20:4ωω6 and docosahexaenoic (C 22:6ω3) acids. The differences could be partly attributed to their variations in the species. Other principal polyunsaturated acid was the omega‐3 fatty acids as shown on the table. The predominant ones were the 20:5ω3 and 22:6ω3 fatty acids. Similar observations were made by Pozo et al. (1992) in their studies on pelagic fish. The abundance of omega‐3‐fatty acids, suggests an additional advantage for the use of the fish species in the formulation of infant foods as they help in the healthy growth and development of the brain, the nervous system and functioning of the eye (Cockburn 1997).

The P/S ratio of these fish species (Table 4) could help reduce the risk of atherosclerosis and coronary heart disease. In western diets the ratio is about 0.6 and it is suggested that increasing it to near 1.0 would be of great health significance (McLennan and Abeywardena 2005).

The low values of the TBARS and the PV (Table 5) are indications of the level of freshness of the fish species and that they may not have undergone any major deterioration in terms of lipid oxidation and its associated reactions.

## Conclusion

Characterization of burrito and the flying gurnard showed that both species are of high nutritional significance in either human food supplements or formulations, as they have a high protein content, good general amino profile, and abundance of polyunsaturated fatty acids. The electrophoretogram of the proteins of these fish species are considered to be species specific and could enable easy identification of the species.

## Conflict of Interest

None declared.
